# From facultative to obligatory parental care: Interspecific variation in offspring dependency on post-hatching care in burying beetles

**DOI:** 10.1038/srep29323

**Published:** 2016-07-05

**Authors:** Alexandra Capodeanu-Nägler, Eva M. Keppner, Heiko Vogel, Manfred Ayasse, Anne-Katrin Eggert, Scott K. Sakaluk, Sandra Steiger

**Affiliations:** 1 Institute of Evolutionary Ecology and Conservation Genomics, University of Ulm, 89081 Ulm, Germany; 2 Department of Entomology, Max-Planck-Institute for Chemical Ecology, 07745 Jena, Germany; 3 Behavior, Ecology, Evolution and Systematics Section, School of Biological Sciences, Illinois State University, Normal IL, USA

## Abstract

Studies on the evolution of parental care have focused primarily on the costs and benefits of parental care and the life-history attributes that favour it. However, once care evolves, offspring in some taxa appear to become increasingly dependent on their parents. Although offspring dependency is a central theme in family life, the evolutionary dynamics leading to it are not fully understood. Beetles of the genus *Nicrophorus* are well known for their elaborate biparental care, including provisioning of their young. By manipulating the occurrence of pre- or post-hatching care, we show that the offspring of three burying beetle species, *N. orbicollis*, *N. pustulatus,* and *N. vespilloides*, show striking variation in their reliance on parental care. Our results demonstrate that this variation within one genus arises through a differential dependency of larvae on parental feeding, but not on pre-hatching care. In *N. pustulatus*, larvae appear to be nutritionally independent of their parents, but in *N. orbicollis*, larvae do not survive in the absence of parental feeding. We consider evolutionary scenarios by which nutritional dependency may have evolved, highlighting the role of brood size regulation via infanticide in this genus.

The evolution of parental care within animal taxa has been addressed in numerous studies focusing on the genetics, physiology and the costs and benefits of care^1^. Parental care has been defined as ‘any parental trait that enhances the fitness of a parent’s offspring, and that is likely to have originated and/or to be currently maintained for this function^2^. In its narrowest sense, parental care includes only those behavioural traits, such as the care of eggs or offspring, which are expressed when young are independent of their parent’s body^3^. The transition from no care to care by one or both parents might seem obvious given that under many conditions, parental care enhances offspring fitness. However, once care evolves, there seems to be an evolutionary trend toward increasing offspring dependency on parental care. Even in arthropods, experimental removal of parents often leads to devastating mortality of offspring due to desiccation, fungal attack or starvation that is not seen in related groups without elaborate parental care^4^,^5^. But the evolutionary forces driving offspring to such severe helplessness remain unclear.

Offspring dependency on parental care might arise for several reasons. One possibility is that dependency of offspring might evolve due to an expansion of the realized niche, i.e. a shift in the species’ distribution into a harsh and inhospitable environment. For example, offspring of the intertidal zone-nesting staphylinid *Bledius spectabilis* are critically dependent on their mother’s behaviour. To prevent flooding and anoxia in their nest, the mother must both close the burrow at high tide and reopen it at low tide^6^,^7^. This scenario assumes, however, that parental care evolved before niche expansion. Recent theory provides another possibility for the evolution of offspring dependence. Parental care often involves intense reciprocal interactions between parents and offspring. Consequently, traits that are involved in such interactions (e.g. parental supply and offspring demand) are expected to co-evolve and may become genetically correlated, ultimately leading to coadaptation of parental and offspring traits^8^,^9^,^10^,^11^. More specifically, a change in the behaviour of parents can alter selection on offspring phenotype which can then result in the further evolution of traits expressed in offspring^12^,^13^,^14^,^15^. In wood roaches of the genus *Salganea* for example, nymphs evolved morphological adaptation of the mouthparts to facilitate the uptake of a specialized liquid diet provided by their mother^7^. Such co-evolved traits certainly enhance the efficiency of nutritional transfer from the mother to the offspring, but at the same time promote offspring dependency. Neonates removed from parental care suffer from high mortality in at least two *Salganea* species, whereas no parental interaction with offspring is known in the closely related genus *Panesthia*^16^, and consequently, the nymphs survive in the absence of parental attendance. This presents one striking example for having distinct patterns of offspring dependency between closely related insects. Nevertheless, the vast majority of previous studies have focused on variation in the form and intensity of care provided by the parents. Whether there can also be variation in the intensity of offspring dependency once care has evolved remains relatively unexplored. To our knowledge, our study is the first to examine the variation in offspring dependency of different species within the same genus of insects, and thus provides an important first step in unravelling the causal basis for variation in offspring dependency.

By adopting a comparative approach, we reveal here remarkable variation in offspring reliance on parental care within one genus of insects, the burying beetles (genus *Nicrophorus*). Burying beetles are well known for exploiting small vertebrate carcasses and providing extensive biparental care to their offspring before and after hatching^17^,^18^,^19^. Before hatching, parents defend the carcass from conspecifics and other insects, bury the carcass and roll it into a ball, removing fur or feathers and applying oral and anal antimicrobial secretions to its surface^17^,^20^,^21^,^22^,^23^,^24^. Females lay their eggs singly in the soil surrounding the carcass. Larvae hatch, crawl to the carcass and aggregate at a small patch of exposed flesh on top of the carrion ball. Parents create this opening in the integument of the carcass surface before or shortly after larval hatching, allowing larvae direct access to the food^25^. By killing and eating some of their first instar larvae, parents frequently reduce the size of their brood^26^. In this way, parents are thought to accomplish an optimal combination of offspring survival and larval size. Post-hatching care in burying beetles consists of continued maintenance and defence of the carcass, as well as regurgitation of pre-digested carrion to the larvae^17^,^18^,^25^. In the most well-studied species to date, *N. vespilloides*, larvae obtain food by begging for pre-digested carrion from their parents as well as by self-feeding directly from the carcass^27^,^28^. From a nutritional point of view, larvae are therefore only partially dependent on their parents, albeit their general survival and growth seems to be significantly improved with parental regurgitation^15^,^25^. A recent study reveals some degree of intraspecific variation in dependency on parental care, and shows that offspring can adapt to changes in parental effort by becoming more independent when selected to evolve in the absence of post-hatching care^29^. Thus, it would not be surprising to find variation in the degree of offspring dependence not only within a species but also between different species. However, aside from an earlier study^30^ suggesting that offspring of different *Nicrophorus* species vary in their degree of offspring dependence, the issue has not been experimentally addressed.

Here, we investigate interspecific variation in the degree of offspring dependence on parental care in three burying beetles species *N. orbicollis*, *N. pustulatus* and *N. vespilloides*. We employed a factorial design in which we experimentally manipulated: (1) the occurrence of pre-hatching care (expressed mainly as preservation of the carcass through antimicrobial secretions) and (2) the occurrence of post-hatching care (expressed mainly through regurgitation of pre-digested carrion and protection of larvae). We evaluated the effect of parental care on the following fitness-related offspring traits in all three species: (1) larval survival to dispersal; (2) larval mass at the time of dispersal; (3) survival from dispersal to eclosion as an adult; (4) adult size. We also assessed the benefit of infanticide within the context of parental care, which is thought to facilitate adjustment of brood size to the size of the carcass^26^. Although a clear test of the benefits of infanticide has been elusive given that removal of parents also leads to a lack of food provisioning, we were able to evaluate the significance of infanticide for offspring fitness opportunistically upon discovering that in one of our three species, parental provisioning does not appear to affect offspring survival and growth.

## Results

### Effects of parental care on larval survival and larval mass (Experiment 1)

**Figure 1:**
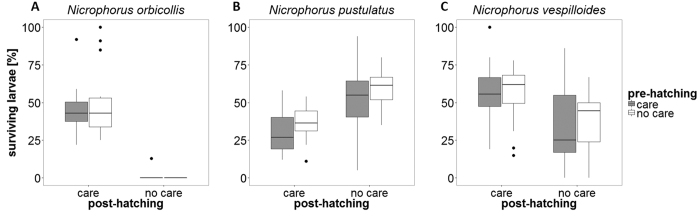
Number of larvae surviving to dispersal relative to the number of eggs laid. (**A**) *N. orbicollis*: n = 65. (**B**) *N. pustulatus*: n = 72. (**C**) *N. vespilloides*: n = 71 (see Supplementary Fig. S1 for the absolute number of larvae surviving to dispersal). Boxplots show median, interquartile range, minimum/maximum range. The dots are values that fall outside the interquartile range (>1.5× interquartile range).

Neither pre-hatching care (GLM with quasi-binomial errors: *F*_1,196_ = 1.43, *P* = 0.23; Table 1, Fig. 1A–C), nor the interaction between pre-hatching and post-hatching care (GLM with quasi-binomial errors: *F*_1,196_ = 0.13, *P* = 0.72), had an effect on larval survival. However, both species (GLM with quasi-binomial errors: *F*_2,196_ = 52.92, *P* < 0.001; Table 1; Fig. 1A–C) and the interaction between species and post-hatching care significantly affected the proportion of larvae surviving (GLM with quasi-binomial errors: *F*_2,196_ = 128.91, *P* < 0.001). The strongest effect of the absence of post-hatching care was observed in *N. orbicollis* (GLM with quasi-binomial errors: *F*_1,63_ = 206.21, *P* < 0.001; Fig. 1A), where larvae survived in only one of 32 broods that did not receive any post-hatching care, but in all 33 broods that did receive post-hatching care. In *N. pustulatus*, in contrast, more larvae survived without than with post-hatching care (GLM with quasi-binomial errors: *F*_1,70_ = 45.28, *P* < 0.001; Fig. 1B). In *N. vespilloides*, larvae survived in 30 of the 33 broods receiving no post-hatching care, but within all surviving broods, survival was significantly higher when parents provided post-hatching care (GLM with quasi-binomial errors: *F*_1,69_ = 14.56, *P* < 0.001; Fig. 1C).

**Figure 2:**
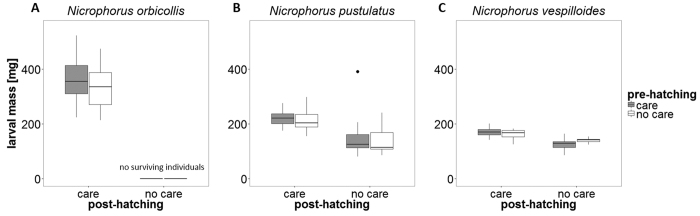
Mass (mg) of larvae surviving to dispersal. (**A**) *N. orbicollis*: n = 33. (**B**) *N. pustulatus*: n = 72. (**C**) *N. vespilloides*: n = 68. Boxplots show median, interquartile range, minimum/maximum range. The dots are values that fall outside the interquartile range (>1.5× interquartile range).

There were significant effects of both species (GLM with Gaussian errors: *F*_1,163_ = 113.93, *P* < 0.001; Table 1 and Fig. 2A–C) and post-hatching care (GLM with Gaussian errors: *F*_1,163_ = 141.10, *P* < 0.001) on larval mass. Because only few larvae survived without post-hatching care in *N. orbicollis*, we could only compare broods receiving full care with broods receiving post-hatching care only. However, there was no difference in larval mass (GLM with Gaussian errors: *F*_1,31_ = 0.17, *P* = 0.68; Fig. 2A). In both *N. pustulatus* and *N. vespilloides*, pre-hatching care had no effect on larval mass at dispersal (GLMs with Gaussian errors: *N. pustulatus*, *F*_1,70_ = 0.42, *P* = 0.52; *N. vespilloides*, *F*_1,66_ = 0.67, *P* = 0.41), whereas post-hatching care did have a significant effect (GLMs with Gaussian errors: *N. pustulatus*, *F*_1,70_ = 38.54, *P* < 0.001; *N. vespilloides*, *F*_1,66_ = 62.78, *P* < 0.001; Fig. 2B,C). In both species, larvae were heavier when reared with parents than in the absence of post-hatching care. In *N. vespilloides*, we also found an interaction effect between pre- and post-hatching care on larval mass (GLM with Gaussian errors: *F*_1,64_ = 6.34, *P* = 0.01; Fig. 2C).

### Effects of parental care on survival to adulthood and pronotum size (Experiment 1)

**Figure 3:**
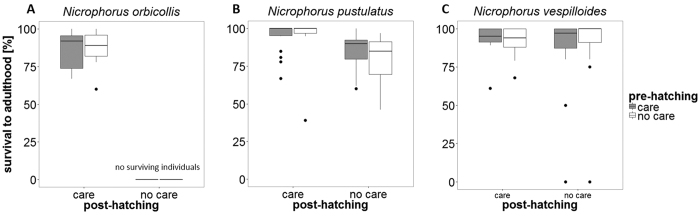
Number of beetles surviving to adulthood relative to the number of larvae that dispersed from the carcass. (**A**) *N. orbicollis*: n = 33. (**B**) *N. pustulatus*: n = 72. (**C**) *N. vespilloides*: n = 66. Boxplots show median, interquartile range, minimum/maximum range. The dots are values that fall outside the interquartile range (>1.5× interquartile range).

Neither pre-hatching care (GLM with quasi-binomial errors: *F*_1,161_ = 0.12, *P* = 0.73; Table 1 and Fig. 3A–C), nor the interaction between pre-hatching and post-hatching care, had any effect on survival to adulthood (GLM with quasi-binomial errors: *F*_1,161_ = 0.21, *P* = 0.65). However, post-hatching care alone (GLM with quasi-binomial errors: *F*_1,161_ = 20.76, *P* < 0.001), and the interaction between species and post-hatching care (GLM with quasi-binomial errors: *F*_1,161_ = 5.41, *P* = 0.02), affected the proportion of larvae surviving to adulthood. We found no difference in survival to adulthood relative to the number of larvae dispersing between broods receiving pre- and post-hatching care and broods receiving post-hatching care only in *N. orbicollis* (GLM with quasi-binomial errors: *F*_1,31_ = 0.01, *P* = 0.91; Fig. 3A). In contrast, survival to adulthood in *N. pustulatus* was significantly affected by post-hatching care (GLM with quasi-binomial errors: *F*_1,70_ = 16.6, *P* < 0.001; Fig. 3B). In this species, more offspring survived to adulthood when receiving post-hatching care than in the absence of post-hatching care. In *N. vespilloides*, the proportion of larvae surviving to adulthood was not affected by pre-hatching or post-hatching care (GLM with quasi-binomial errors: *F*_1,64_ = 1.29, *P* = 0.26 ; Fig. 3C).

**Figure 4:**
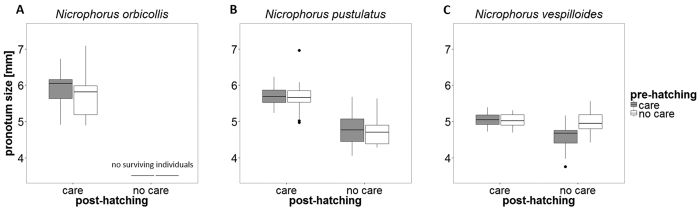
Pronotum size (mm) of offspring that eclosed. (**A**) *N. orbicollis*: n = 33. (**B**) *N. pustulatus*: n = 72. (**C**) *N. vespilloides*: n = 66. Boxplots show median, interquartile range, minimum/maximum range. The dots are values that fall outside the interquartile range (>1.5 × interquartile range).

Both species (GLM with Gaussian errors: *F*_2,160_ = 23.80, *P* < 0.001; Table 1; Fig. 4A–C) and post-hatching care (GLM with Gaussian errors: *F*_1,160_ = 98.47, *P* < 0.001), and the interaction between species and post-hatching care (GLM with Gaussian errors: *F*_1,160_ = 16.28, *P* < 0.001), influenced adult pronotum size. In *N. orbicollis*, we found no difference in pronotum size between beetles that were reared in broods receiving both pre- and post-hatching care and broods receiving post-hatching care only (GLM with Gaussian errors: *F*_1,31_ = 0.81, *P* = 0.38; Fig. 4A). In *N. pustulatus*, only post-hatching care significantly affected pronotum size, i.e. beetles from broods that received post-hatching care were larger than beetles from broods that did not receive post-hatching care (GLM with Gaussian errors: *F*_1,69_ = 96.55, *P* < 0.001; Fig. 4B). In *N. vespilloides*, pre-hatching (GLM with Gaussian errors: *F*_1,63_ = 4.86, *P* = 0.03; Fig. 4C) and post-hatching care (GLM with Gaussian errors: *F*_1,63_ = 12.62, *P* < 0.001; Fig. 4C), and the interaction of both forms of care (GLM with Gaussian errors: *F*_1,61_ = 10.36, *P* = 0.00; Fig. 4C) all had an effect on the beetles’ pronotum size. In this species, pronotum size was similar in beetles from broods that received post-hatching care and broods that received no care at all. In contrast, beetles from broods that received pre-hatching care only were smaller than beetles from the other treatments. There was no difference across treatments in female and male pronotum size in any species (GLMs with Gaussian errors: *N. orbicollis*, *F*_1,64_ = 0.11, *P* = 0.75; *N. pustulatus*, *F*_1,177_ = 0.11, *P* = 0.74; *N. vespilloides*, *F*_1,156_ = 2.37, *P* = 0.13; Supplementary Fig. S2).

### The effect of parental brood size regulation on offspring growth in *N. pustulatus* (Experiment 2)

Upon manipulating brood size of *N. pustulatus*, we found that post-hatching care no longer had an effect on larval mass (GLM with Gaussian errors: *F*_1,44_ = 1.24, *P* = 0.27); there was no difference in the mass of larvae receiving full parental care and those receiving pre-hatching care only. In addition, there was no difference in pronotum size between beetles from broods that received full parental care and beetles from broods that received pre-hatching care only (GLM with Gaussian errors: *F*_1,44_ = 3.57, *P* = 0.07). We found no significant difference in survival to adulthood between treatments (GLM with quasi-binomial errors: *F*_1,44_ = 0.34, *P* = 0.56). Although parents of some broods still regulated the number of larvae, we found no difference in the proportion of larvae surviving between the two treatments (GLM with quasi-binomial errors: *F*_1,44_ = 3.83, *P* = 0.06).

## Discussion

Our study revealed that offspring of the three burying beetle species, *N. orbicollis*, *N. pustulatus*, and *N. vespilloides*, differ greatly in their reliance on parental care. Whereas parental care is facultative in *N. pustulatus* and *N. vespilloides*, *N. orbicollis* appears to be a beetle with obligatory parental care, as offspring do not survive in the absence of parents. Our results also revealed that this striking interspecific variation in offspring reliance on parental care is caused by a differential dependency on the post-hatching, but not pre-hatching, component of care. In addition, our study directly documented the adaptive value of infanticidal brood-size regulation in burying beetles. By killing some of their own larvae, parents improve the growth and survival of the remaining brood. Below we elaborate on the selective factors leading to these interspecific differences, the role of filial cannibalism, and the evolutionary transition to parental care in burying beetles.

We found that post-hatching care affected larval survival or growth of all three species, whereas pre-hatching care, which in our laboratory environment was manifest primarily in the preservation of the carcass via antimicrobial secretions, did not have any significant effect on offspring performance. In the field, pre-hatching care would also include burial of the carcass, which can reasonably be presumed to significantly lower the risk of losing the carcass to vertebrate scavengers or competing invertebrates in all species of burying beetles. Although oral and anal secretions applied to the carcass influence larval development in *N. vespilloides*^24^, our study suggests that post-hatching care is more important to offspring fitness than carcass preparation, at least under the conditions established in this study. Parents regularly apply anal secretions containing lysozyme to the carcass^21^,^24^,^31^, and the resulting lytic activity aids in the preservation of the carcass (for *N. vespilloides*^24^,^32^; for *N. orbicollis*^23^). Previous studies have shown that this form of pre-hatching care is beneficial for larval development, but a benefit was found only when carcasses had been aged for at least one week before being given to breeding adults^32^. The starting point for our experiment was a fresh carcass, and the larvae that did not receive any pre-hatching care were provided with a carcass that had aged according to the respective pre-hatching care period, i.e. 80 h in *N. pustulatus* and 70 h in *N. vespilloides*. Using a fresh carcass might explain why pre-hatching care in our study had no effect on offspring survival, mass or adult size in any of the three species. However, in nature, pre-hatching care could still have an effect on offspring performance. First, the microbial community in the beetles’ natural environment likely poses a greater challenge than the one presented in the laboratory. Second, by preserving the carcass, burying beetles are better able to control decomposition and prevent olfactory cues from escaping from the carcass. By masking the cadaveric volatiles that are released from the nest, beetles can hinder potential competitors from being attracted to the carcass^33^,^34^. We note that in the case of *N. vespilloides*, larvae actually grew larger when receiving no care at all than when carcasses had been prepared. This result is puzzling and could argue against the evolution of pre-hatching care. We think, however, that the application of antimicrobial secretions to the carcass is certainly beneficial and hinders the deterioration of the carcass from microbial decomposition^32^. Nonetheless, the presence of the parents during the pre-hatching period likely also reduces the nutritional value of the carcass as they feed on it for their own maintenance^18^,^35^. Another factor that could account for this unexpected result is that microbial activity in the absence of parental provisioning might be beneficial to larvae if microbial biodegradation facilitates the uptake and digestion of the carrion by the developing larvae, particularly if larvae are poor self-feeders.

The effect of post-hatching care in our study was quite evident and it varied greatly between the three species. Eliminating post-hatching care in *N. orbicollis* resulted in devastating mortality, such that few larvae survived. In contrast, larval survival of *N. pustulatus* was actually higher in the absence of post-hatching care than when parents were present, a finding that is consistent with the results of Rauter and Moore^36^, who also found that fewer larvae left the carcass when a parent was present. The reliance of *N. vespilloides* larvae on post-hatching care appears to be intermediate, as offspring survival in the absence of post-hatching care was better than in *N. orbicollis*, but worse than in *N. pustulatus*. In general, post-hatching care consists of: (1) continued maintenance of the carcass and protection of the larvae against microorganisms, (2) defence of the carcass and larvae against predators and competitors, and (3) feeding larvae with pre-digested carrion. However, as the experiment was conducted under laboratory conditions, defence against predators and competitors obviously did not play a role in our experiments. The lack of a difference in offspring survival on carcasses treated with antimicrobial secretions and untreated ones, suggests that protection against microbes was not an essential element of post-hatching care, although the microbial community in the beetles’ natural environment might pose a greater challenge than the one in the laboratory. Collectively, our experiments suggest that a differential dependency on parental feeding was the critical factor causing the observed differences in survival between the three species. These results are consistent with the findings of Trumbo^30^, who compared the ability of larvae to survive on chicken liver in the absence of parental feeding in five North American species, and found that *N. orbicollis* larvae failed to survive to the second instar, while *N. pustulatus* larvae developed normally in the absence of parental feeding. Although larvae of all three species are able to self-feed on the carcass (*N. orbicollis*^37^; *N. pustulatus*^30^,^36^; *N. vespilloides*^25^), the ability to self-feed in *N. vespilloides* depends on the age of the larvae, with younger larvae being less efficient self-feeders than older ones^25^,^28^. *N. orbicollis* larvae might not be able to self-feed at all until they reach a certain age or mass, or alternatively, self-feeding might not be sufficient to sustain the rapid growth required in the largest of the three species^30^. In contrast, our results suggest that *N. pustulatus* larvae become efficient self-feeders shortly after hatching, and that parental regurgitation does not improve survival and mass of the larvae, although it might have other positive effects. For example, Rauter and Moore^36^ found that development of larvae was accelerated in the presence of maternal care.

Our observation that even more *N. pustulatus* larvae survived in the absence than in the presence of post-hatching care might seem counterintuitive, but this result can be easily explained by the absence of parental-brood size adjustment when post-hatching care was prevented. Burying beetles are known to lay excess eggs as insurance against hatching failure^26^,^38^. When hatching success is high and most larvae reach the carcass, parents kill some of the larvae to match brood size to the carcass size, i.e. the availability of food. In the presence of parental care, siblings compete fiercely for carrion which results in a negative correlation between the density of larvae and larval mass^38^,^39^,^40^. Thus, it has been hypothesised that the function of this killing is to reduce larval competition and starvation of offspring^26^,^38^. Our second experiment clearly demonstrated that the cause of the lower larval mass at dispersal in *N. pustulatus* in the absence of post-hatching care was not the lack of parental provisioning per se, as was the case in *N. vespilloides*, but rather, larval competition for food caused by the absence of parental brood reduction. By experimentally reducing the size of the broods that subsequently received either full care or pre-hatching care only, we were able to show that the presence of post-hatching care no longer had an effect on larval mass or adult pronotum size. This shows that the larvae of some species, such as *N. pustulatus*, develop equally well with and without parental regurgitation when food is not in short supply. Moreover, the results of our study support Bartlett’s^26^ hypothesis that parental infanticide reduces larval competition for food. When parents were allowed to regulate the size of their brood, fewer larvae survived, but individual larval masses and subsequent adult sizes were higher than when brood reduction was blocked in *N. pustulatus*. Furthermore, the percentage of larvae that survived to adulthood was higher when parents were allowed to engage in filial cannibalism, than when such infanticide was prevented. This suggests that due to the high level of competition among larvae reared without parental interference, a portion of the brood did not acquire sufficient resources for pupation or eclosion. Infanticide in burying beetles can therefore be considered an adaptive strategy by which parents optimize the number of surviving young.

Trumbo^30^ proposed that *Nicrophorus* evolved from an ancestral species that was not dependent on parental feeding, as larvae in the sister genus *Ptomascopus* do not appear to require parental assistance in processing carrion. Brood guarding, the defence of the carcass and the offspring against conspecific or congeneric competitors and predators, may have been the primary force for a prolonged residency by one or both parents^18^. Our study lends some support to this hypothesis because although *N. pustulatus* larvae are nutritionally independent of their parents, parents nevertheless stay with their brood for a prolonged period^41^. Our data might also suggest that infanticidal brood regulation preceded the evolution of offspring feeding because in *N. pustulatus*, brood regulation is of higher importance for offspring fitness than parental provisioning. It seems likely that once the period of residency of breeding beetles evolved to encompass the hatching period, females were afforded the opportunity to increase clutch size as insurance for hatching failure, and then afterwards could adjust brood size according to the amount of available carrion. Following from this, clutch size regulation, which also occurs in *Ptomascopus*, is likely to have preceded both brood regulation and offspring feeding. However, a larger comparative study would be needed to test this hypothesis.

Our study ultimately raises the question as to why parental provisioning evolved, and why the larvae of some species have become nutritionally dependent and lost their ability to self-feed on the carcass. Eggert and Müller^18^ suggested that parental feeding may have accelerated consumption of the carcass, thereby decreasing the duration of care and reducing the vulnerability of the brood to intruders. A recent model^42^ suggests that parental provisioning only evolves if it is more efficient than offspring are at self-feeding, or when parents are more efficient at feeding than guarding the offspring against predators. This model also predicts that the evolution of food provisioning promotes a mutual reinforcement between parental feeding and sibling competition, resulting in a unidirectional trend from no parental food provisioning to full parental food provisioning. Once feeding evolved, coadaptation between offspring and parental traits might have led to a loss of traits, or more precisely, decelerated the development of traits necessary for self-feeding so that they were only expressed later in life. As parents took over the task of digesting the carcass, relaxed selection on the production of digestive enzymes in larvae might have led to an increase in nutritional dependency. Surprisingly, a recent study using a cross-fostering technique in the two species *N. orbicollis* and *N. vespilloides* found no interaction between the care-giving species and the recipient species in their effects on offspring development or mass^43^. Here, the authors suggest that rather than representing a species-specific coadaptation between parents and offspring, maternal effects resulting from parental care appear to be generalized with respect to their value to offspring within this genus^43^. It is known that burying beetle parents adjust the time spent provisioning to the amount of offspring begging^27^,^44^. Although parents can influence sibling competition within a brood^45^,^46^, offspring are able to control food allocation^28^. In relation to our results, this could imply that larvae of different species vary in their amount of begging and that parents have adapted to respond to this begging signal even though this adaptation might not be species-specific.

A second route may have involved co-evolution of two maternal traits, egg size and parental feeding. Two previous studies suggest that parental provisioning in *N. vespilloides* can compensate for a reduction in egg size^35^,^47^. Hence, selection on parental feeding might have led to a reduced investment of resources into eggs, and consequently to underdeveloped and more altricial hatchlings. In line with this, a comparative study on three shield bug species showed that the species with the largest clutch size and the smallest eggs cared for their young the longest^48^. However, *N. pustulatus* females lay by far the smallest eggs of the three species (pers. obs.), so that this alternative evolutionary scenario is unlikely to account for an increase in offspring dependency.

Finally, a definitive causal explanation for the remarkable interspecific variation in offspring dependency on parental provisioning documented in this study remains elusive. An understanding of this variation probably begins with a consideration of the life-history strategies observed in different burying beetle species. For example, larvae of species rearing large broods are likely to be selected to maintain their independence, because parents cannot attend to each larva as well as parents of smaller broods^30^. In line with this, *N. pustulatus* females produced by far the largest clutch of the three species examined. Apart from their ability to bury and prepare vertebrate carcasses as a food source for their offspring^30^,^36^, beetles of this species are known to exploit snake eggs (e.g. of the black rat snake *Elaphe obsoleta*)^49^,^50^. This host shift is thought to be a selective force driving the evolution of the large clutch size in this species, as an adaptation to exploit the unusual mass of a clumped resource, eggs in the nests of snakes.

In a wider context, care in birds and mammals is usually obligate, and offspring cannot survive without some care early in their life. Offspring of altricial birds, for example, are unable to walk or fly. Thus, nesting far from the ground makes offspring necessarily dependent on parental food provisioning. In mammals, females always provide care in form of milk and most rodents, cats or dogs have highly dependent young that not only need to be fed, but also to be warmed and protected^1^. In contrast, insects show a broad spectrum of care behaviours, ranging from no care to facultative and obligate parental care. For example, earwig species vary greatly in their degree of maternal care, with some provisioning and defending the nymphs into the second instar and beyond, others ending care soon after hatching, and some assisting only in eclosion^7^. Although earwig larvae can survive without tending mothers, removal experiments have shown that maternal care clearly enhances larval survival^51^. There are other examples found in shield bugs, leaf or dung beetles where the duration of maternal care differs between species of one genus and seems often to be linked to different reproductive strategies or diverse habitats^7^. However, those studies only address the plasticity of care behaviours from the parents’ perspective, but neglect the consequences that may differ for dependent offspring. Our study provides new insights for the understanding of offspring dependency in *Nicrophorus* and presents a unique opportunity to further unravel the dynamics of parental care evolution. We have shown that distinct variation in offspring dependency can occur within a single genus of insects that have been reared on the same breeding resource in a constant environment. The degree of parental care and food provisioning seems to influence traits expressed in offspring and the nutritional dependence associated with these traits. Thus, our results strongly suggest that offspring dependency in burying beetles has not arisen due to an expansion of an ecological niche, but more likely to a coadaptation of parent and offspring traits. A more expansive comparative study, one that accounts for phylogenetic relationships within the group^52^, could yield even greater insight in the evolutionary transitions leading from self-sufficient offspring to young that are wholly dependent on parental care early in their development, particularly the co-evolutionary dynamics of parental provisioning and nutritional dependency of offspring.

## Materials and Methods

### Origin and maintenance of experimental animals

Experimental *N. vespilloides* were descendants of beetles collected from carrion-baited pitfall traps in a forest near Ulm, Germany (48°25′03″N, 9°57′45″E). Cultures of *N. pustulatus* and *N. orbicollis* were established at Ulm University in August 2014 from outbred colonies maintained in the Institute of Zoology at the University of Freiburg, Germany. Individuals of both species originated from beetles caught in baited pitfall traps near Lexington, Illinois, USA (40°39′57″N, 88°53′49″W). All beetles were maintained in climate chambers at 20 °C under a 16:8 h light:dark cycle. Before the experiments, groups of up to five adults of the same sex and family of each species were kept in small plastic containers (10 × 10 cm and 6 cm high) filled with moist peat and fed freshly decapitated mealworms ad libitum twice a week.

### Experiment 1: The importance of pre-and post-hatching parental care for offspring survival and growth

**Figure 5:**
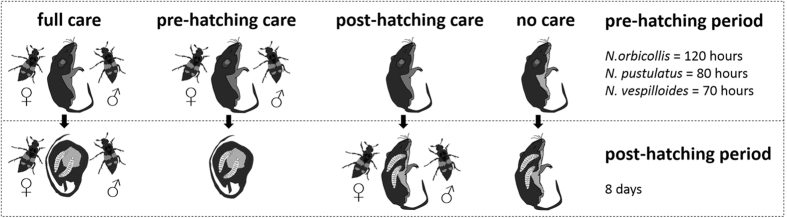
Schematic diagram of the experimental procedure. In the “full care” treatment, parents were allowed to provide both pre-hatching and post-hatching care. In the “pre-hatching care” treatment, parents were allowed to prepare the carcass but were removed from the carcass as soon as the larvae hatched. In the “post-hatching care” treatment, parents were only allowed to provide care after the larvae had hatched. In the “no care” treatment, parents were not allowed to provide pre-hatching or post-hatching care.

To measure interspecific variation in the dependency of offspring on parental care, and to determine whether this variation is caused by a reliance on pre-hatching care, post-hatching care, or both, we established four treatments in which we simultaneously manipulated the occurrence of pre- and post-hatching care in each species (Fig. 5). Non-sibling pairs of beetles were randomly assigned to the different groups and reproduction was induced by providing them with a 20 g (±3 g) thawed mouse carcass (Frostfutter.de–B.A.F Group GmbH, Germany). In the case of the nocturnal species, *N. pustulatus* and *N. orbicollis*, mice were provided during the dark portion of the photoperiod. After the egg-laying period, but before the hatching of larvae (*N. orbicollis*: 72 h; *N. pustulatus*: 72 h; *N. vespilloides*: 48 h), parents were transferred to new plastic containers and old containers were searched for eggs to determine clutch size in all treatments. After clutch size had been determined, the eggs were returned to their original container and provided with a carcass and/or their parents according to the treatment group to which they had been assigned. To simulate underground conditions, containers were stored in the dark during the remainder of the experiment.

In the “full care” treatment, parents were allowed to provide both pre-hatching and post-hatching care (*N. orbicollis*: n = 16; *N. pustulatus*: n = 18; *N. vespilloides*: n = 18). In the “pre-hatching care” treatment, parents were allowed to prepare the carcass and treat it with antimicrobial secretions, but were removed from the carcass as soon as the larvae hatched (*N. orbicollis*: n = 16; *N. pustulatus*: n = 18; *N. vespilloides*: n = 17). In the “post-hatching care” treatment, parents were only allowed to provide care after the larvae had hatched (*N. orbicollis*: n = 17; *N. pustulatus*: n = 18; *N. vespilloides*: n = 20). As a carcass is required to initiate egg laying and this carcass is inevitably prepared by the parents, the original prepared carcass was replaced with a new unprepared carcass at the time the larvae hatched. The parents and larvae were given an unprepared dead mouse that had been thawed and kept in a box with soil in the laboratory at 20 °C for a time period equivalent to the species-specific pre-hatching care period (*N. orbicollis*: 120 h; *N. pustulatus*: 80 h; *N. vespilloides*: 70 h). In the “no care” treatment, parents were not allowed to provide pre-hatching or post-hatching care (*N. orbicollis*: n = 16; *N. pustulatus*: n = 18; *N. vespilloides*: n = 16). Here, the same protocol as in the post-hatching care treatment was applied except that parents were removed at the time the prepared carcass was replaced with an unprepared carcass. As it was not our intention to analyse the effect of the opening that parents chew into the carcass before the larvae arrive, an opening was artificially created by cutting a hole into the skin of the mouse using scissors in all treatment groups^25^,^32^. This procedure afforded larvae the possibility to access the carrion without the barrier presented by the skin of the carcass, thereby facilitating self-feeding.

As soon as larvae of each brood left the carcass for pupation (i.e. approx. 10 days after carcass provisioning), they were counted, weighed and transferred to new plastic containers with moist peat that filled about two-thirds of the box. After eclosion, the number of surviving adults was determined for each brood and their pronotum size measured.

### Experiment 2: The effect of parental brood size regulation on offspring growth in *N. pustulatus*

In *N. pustulatus*, larval mass at dispersal was higher in the presence than in the absence of post-hatching care (see Fig. 2B). However, because brood size was lower in the presence of post-hatching care due to parental brood-size adjustments, two factors could have been responsible for the higher larval mass: parental brood-size adjustment (and the lower number of larvae resulting from it), or parental feeding. To disentangle the influence of the two factors, we conducted an additional experiment in which we manipulated brood size in this species to simulate parental brood-size adjustments. Pairs of beetles in either the “full care” treatment (n = 21) or the “pre-hatching care” treatment (n = 25) were provided with a thawed dead mouse (20 g ± 3 g) for reproduction. After 72 h, both the parents and the carcass were transferred into new plastic containers filled with soil, and old containers were checked every four hours for the presence of newly hatched larvae. To create broods of mixed parentage, all newly hatched larvae were pooled every four hours and maintained in a Petri dish with moist filter paper at 4 °C until used. In the full care treatment, parents and their associated carcass were again transferred to a new container before being provided with a randomly chosen brood of twelve larvae. As females exhibit temporally-based kin discrimination in which they kill any larvae arriving on the carcass before their own eggs would have hatched, but accept larvae that arrive after their own eggs have begun to hatch^53^, we only provided pairs with larvae after their own larvae had begun to hatch. In the pre-hatching care treatment, twelve larvae were added on top of the prepared carcass in the absence of parents. We opted for an experimental brood size of twelve larvae as *N. pustulatus* parents rarely adjusted the size of their brood below this number in experiment 1. To avoid any disturbance to experimental pairs, all transfers were performed in the dark under red light. As in the previous experiment, we created an opening into the prepared carcasses of both treatment groups.

Similar to experiment 1, larvae from each brood that left the carcass for pupation were counted, weighed and transferred into new plastic containers with moist peat. All newly eclosed adults were counted and pronotum size was measured for each treatment.

### Statistical analyses

All data were analysed and plotted using R version 3.1.2 (R Core Team 2014). The experimental procedure of experiment 1 corresponds to a 3 × 2 × 2 factorial design, in which pre-hatching (no care or care) and post-hatching parental care (no care or care) was manipulated in each of the three species. Hence, we performed generalised linear models (GLMs) with species, pre-hatching care, post-hatching care and species*pre-hatching*post-hatching care as fixed factors, and the proportion of larvae surviving, mean larval mass per brood, mean pronotum size and the proportion of eclosing beetles as dependent variables. We additionally performed GLMs within each species with the dependent variables mentioned above. As only few larvae survived without post-hatching care in *N. orbicollis*, mean larval mass, mean pronotum size and the proportion of eclosing beetles could only be compared between broods that received full care and those that only received post-hatching care. For the overall model as well as for the separate species models, we calculated the proportion of larvae surviving to dispersal relative to the absolute number of eggs laid for each brood as clutch size differed between the three species (GLM with quasi-Poisson errors: *F*_2,276_ = 217.21, *P* < 0.0001; median, 1. quartile, 3. quartile: *N. orbicollis*: 29, 22, 35; *N. pustulatus*: 67, 53, 81; *N. vespilloides*: 26, 14.5, 36). Assuming that all eggs had hatched successfully and neglecting unfertilized or non-viable eggs, we created a two-column response vector consisting of the number of positives (i.e. number of larvae that survived to dispersal) and the number of negatives (i.e. number of larvae that did not survive to dispersal) joined together by the function *cbind* in R 3.1.2. The same applies to the number of offspring surviving to adulthood in the overall model as well as in the separate species models, where we created the response vector with positives (i.e. number of eclosing adults) and negatives (i.e. number of dispersing larvae that failed to eclose) from the total number of larvae surviving to dispersal for each brood. We then applied GLMs with quasi-binomial distribution to correct for overdispersion in our data. GLMs for mean larval mass and mean pronotum size were calculated with Gaussian errors.

## Additional Information

**How to cite this article**: Capodeanu-Nägler, A. *et al*. From facultative to obligatory parental care: Interspecific variation in offspring dependency on post-hatching care in burying beetles. *Sci. Rep.* **6**, 29323; doi: 10.1038/srep29323 (2016).

## Supplementary Material

Supplementary Information

## Figures and Tables

**Table 1:** Effects of parental care on fitness-related offspring traits across species.

Factor	Larval survival		Larval mass		Survival to adulthood		Pronotum size	
	*F*	*P*	*F*	*P*	*F*	*P*	*F*	*P*
post-hatching	1.62	0.20	141.10	**<0.001**	20.76	**<0.001**	98.47	**<0.001**
pre-hatching	1.43	0.23	0.48	0.49	0.12	0.73	0.15	0.70
species	52.92	**<0.001**	113.93	**<0.001**	1.68	0.19	23.80	**<0.001**
post-hatching*pre-hatching	0.13	0.72	0.20	0.66	0.21	0.65	1.13	0.29
post-hatching*species	128.91	**<0.001**	3.61	0.06	5.41	**0.02**	16.28	**<0.001**
pre-hatching*species	1.32	0.27	0.29	0.75	0.08	0.92	2.16	0.12
post-hatching*pre-hatching*species	0.73	0.48	0.50	0.48	0.80	0.37	3.59	0.06

Results of GLMs of the effect of species (*N. orbicollis*, *N. pustulatus*, *N. vespilloides*), pre-hatching care (no care or care), post-hatching care (no care or care) and the interactions of factors on larval survival, larval mass, survival to adulthood and pronotum size. Significant p-values are typed in bold.
