# Supplementary material for: From facultative to obligatory parental care: Interspecific variation in offspring dependency on post-hatching care in burying beetles
Source: Sci Rep. 2016 Jul 5;6:29323. doi: 10.1038/srep29323 (PMC4932505; doi:10.1038/srep29323)
Supplement: Supplementary Information [file srep29323-s1.pdf]

Supporting Information

From facultative to obligatory parental care: Interspecific variation in offspring dependency on post-hatching care in burying beetles

Alexandra Capodeanu-Nägler, Eva M. Keppner, Heiko Vogel, Manfred Ayasse, Anne-Katrin Eggert, Scott K. Sakaluk, Sandra Steiger

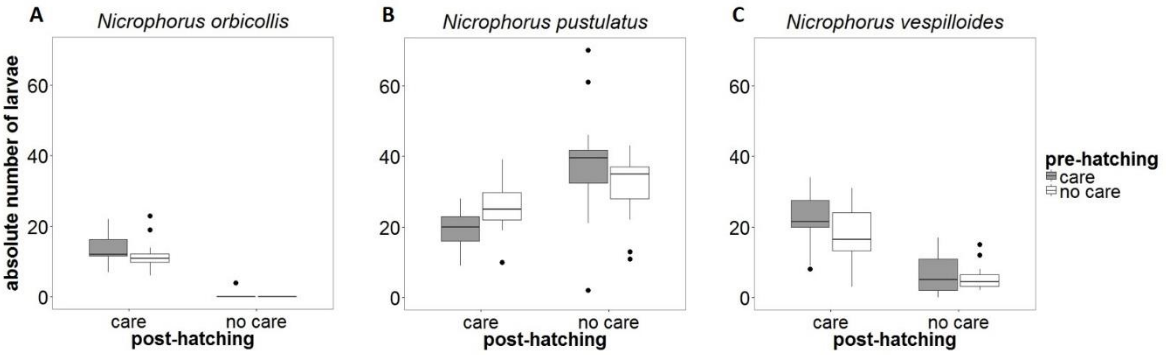

Supplementary Figure S1. Absolute number of larvae surviving to dispersal. (A) *N. orbicollis*: n = 65. (B) *N. pustulatus*: n = 72. (C) *N. vespilloides*: n = 71. Boxplots show median, interquartile range, minimum/maximum range. The dots are values that fall outside the interquartile range (> 1.5 x interquartile range).

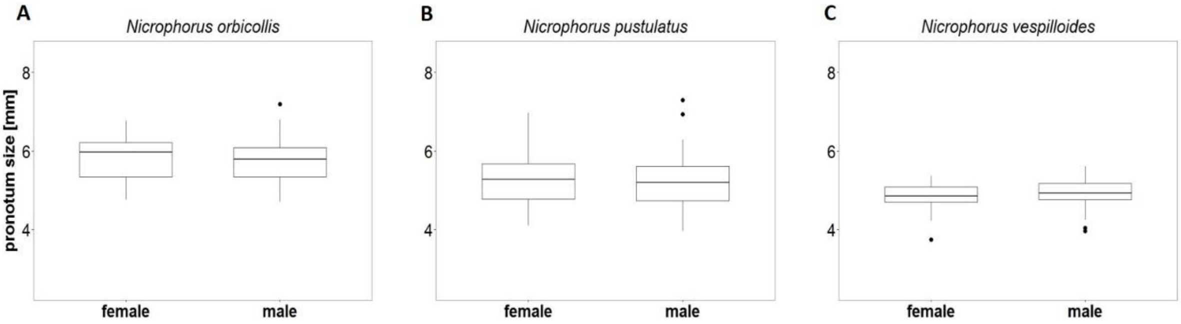

Supplementary Figure S2. Pronotum size (mm) of females and males. (A) *N. orbicollis*: n = 33. (B) *N. pustulatus*: n = 72. (C) *N. vespilloides*: n = 66. Boxplots show median, interquartile range, minimum/maximum range. The dots are values that fall outside the interquartile range (> 1.5 x interquartile range).
